# Skeletal muscle mass estimation in Brazilian Jiu-Jitsu athletes: validation of predictive equations

**DOI:** 10.3389/fnut.2025.1595259

**Published:** 2025-07-18

**Authors:** Alex Ojeda-Aravena, Eduardo Báez-San Marín, Xurxo Dopico-Calvo, Mauricio Cresp-Barría, Jorge Olivares-Arancibia, Jairo Azócar-Gallardo

**Affiliations:** ^1^Departamento de Ciencias de la Actividad Física, Universidad de Los Lagos, Osorno, Chile; ^2^Laboratorio de Fisiología del Ejercicio y Rendimiento Deportivo, Facultad de Ciencias de la Actividad Física y del Deporte, Universidad de Playa Ancha, Valparaíso, Chile; ^3^Facultad de Ciencias de la Vida, Carrera de Entrenador Deportivo, Universidad Viña del Mar, Viña del Mar, Chile; ^4^Performance and Health Group, Department of Physical Education and Sport, Universidade da Coruña, A Coruña, Spain; ^5^Department of Innovation and Education, Faculty of Education, Catholic University of Temuco Chile, Temuco, Chile; ^6^AFySE Group, Research in Physical Activity and School Health, School of Physical Education, Faculty of Education, Universidad de las Américas, Santiago, Chile; ^7^Programa de Investigación en Deporte, Sociedad y Buen Vivir (DSBv), Universidad de Los Lagos, Osorno, Chile; ^8^Departamento de Ciencias de la Actividad Física, Universidad de Los Lagos, Puerto Montt, Chile

**Keywords:** skeletal muscle mass, Brazilian Jiu-Jitsu, predictive equations, body composition assessment, dual-energy X-ray absorptiometry

## Abstract

Accurate estimation of skeletal muscle mass (SMM) is important for body composition assessment in Brazilian Jiu-Jitsu (BJJ) athletes owing to body mass classification and force production implications. This study compared the validity, reliability, and agreement of three predictive equations—Kim, McCarthy, and Sagayama—for estimating total SMM (expressed in kilograms) in male BJJ athletes. Twenty-two male BJJ athletes (mean age: 33.1 ± 7.5 years; body mass: 78.4 ± 9.6 kg; height: 171.8 ± 6.4 cm) underwent DXA-derived body composition analysis. SMM was estimated using the Kim, McCarthy, and Sagayama equations. Statistical analyses included repeated-measures ANOVA, stepwise linear regression, Pearson's correlation, intraclass correlation coefficient (ICC), coefficient of variation (CV%), and Bland-Altman plots. The mean SMM estimated by the Kim equation was 28.95 ± 4.92 kg (95% CI: 26.89–31.00 kg), by the McCarthy equation, 27.39 ± 4.96 kg (95% CI: 25.32–29.47 kg), and by the Sagayama equation, 27.72 ± 3.71 kg (95% CI: 26.16–29.27 kg). The Kim equation yielded significantly higher SMM values than McCarthy (mean difference = 1.55 kg, *p* < 0.0001), while Sagayama and McCarthy did not differ significantly. Stepwise regression identified the Kim equation as a strong predictor of Sagayama SMM values (*R* = 0.851; *R*^2^ = 0.724; RMSE = 2.0 kg; *F*_1, 20_ = 52.369; *p* < 0.001), although with proportional underestimation (slope = 0.642). Reliability was acceptable for all equations (ICC > 0.79), and the Sagayama equation demonstrated the lowest CV% (13.4%, 95% CI: 9.44%−17.36%). Bland–Altman analysis revealed systematic biases, particularly for the Kim equation. All three equations provided accurate validity and reliability for estimating absolute SMM (kg) in BJJ athletes. However, the McCarthy and Sagayama equations showed less bias and greater agreement by DXA, supporting their use for accurate quantification of SMM in this population. Their validation with magnetic resonance imaging is needed.

## 1 Introduction

Skeletal muscle mass (SMM) is a fundamental physiological indicator of body composition (BC) that directly affects metabolic health, athletic performance, and disease prevention (1, 2). Striated skeletal muscle is considered a malable organ in response to environmental stimuli, underscoring its importance in athletic populations (3, 4). SMM is typically expressed in kilograms, grams, or as a percentage of the total body mass (1).

Currently, magnetic resonance imaging (MRI) is the gold standard for determining SMM volume, allowing conversion to mass using a standard density of 1.04 kg (5). However, this technique has practical limitations owing to its high cost and limited accessibility to the general population. Consequently, dual-energy X-ray absorptiometry (DXA) has been established as a viable and reliable alternative for estimating SMM (1). DXA enables the quantification of both Free-Fat Mass (FFM) and appendicular lean tissue (ALM), which is a more specific proxy for SMM (6, 7).

Despite its utility, DXA-derived fat-free mass (FFM) tends to overestimate SMM because it includes components such as total body water and bone mineral content (BMC). To address these limitations, focusing on appendicular lean tissue, which excludes bone mineral content, provides a more accurate estimation of SMM (6). However, both FFM and ALM include intermuscular fat, potentially leading to an overestimation of SMM (6).

In Brazilian Jiu-Jitsu (BJJ) athletes, SMM has historically been estimated using proxies derived from bioimpedance and anthropometry, such as FFM and fat-free mass index (FFMI), as well as SMM-specific equations (8–11). However, these methods have important limitations, as they assume that all FFM correspond to SMM without distinguishing intramuscular fat infiltration from other non-fat components. This impedes systematic comparisons between studies and may lead to inaccurate estimates (5).

Several predictive equations have been developed and validated using MRI and DXA to improve precision. Kim et al. (5) proposed models for different age groups. Recently, McCarthy et al. (6) developed equations in a representative sample of adults, allowing the estimation of SMM at both the total and regional levels. Despite these advances, the validation of such models has primarily been performed in the general population, with limited evaluation of their applicability in athletic populations, particularly in sports with high muscular demands such as BJJ (12).

To date, only Sagayama et al. (13) have validated predictive equations in combat sports athletes (judokas and wrestlers), demonstrating that inclusion of the trunk/ALM ratio improves estimation accuracy in physically active individuals. However, the generalizability of these findings to other disciplines such as BJJ remains insufficiently explored.

Given the importance of SMM in athletic performance, injury prevention, and overall athletic health, accurate and reliable methods tailored to the specific physical demands of BJJ are essential (12, 14, 15). Validating the existing predictive equations in this population would optimize the accuracy of SMM assessment and enable the development of more effective and individualized training and nutritional strategies.

Accordingly, the present study aimed to assess the validity, reliability, and agreement of different predictive equations for estimating SMM in BJJ athletes. We hypothesized that the equation proposed by Sagayama et al. (13) would demonstrate superior validity compared with those of Kim et al. (5) and McCarthy et al. (6) SMM equations, as it was previously validated in combat athletes and incorporates variables of greater morpho-functional relevance for this population.

## 2 Methods and procedures

### 2.1 Participants

Twenty-two male BJJ athletes (age: 33.43 ± 7.89 years [95% CI: 30.13–36.72 years], height: 171.81 ± 6.36 cm [95% CI: 169.15–174.47 cm], body mass: 77.67 ± 9.26 kg [95% CI: 73.80–81.54 kg], BJJ training experience: 7.71 ± 5.19 years [95% CI: 5.54–9.88 years]; weekly training frequency: 3.50 ± 1.01 sessions [95% CI: 3.07–3.92 sessions]; weekly training volume: 310.91 ± 95.11 min [95% CI: 271.16–350.65 min]) were enrolled in this study.

The inclusion criteria were as follows: (1) athletes who trained a minimum of three times per week, (2) had at least 2 years of continuous BJJ training, (3) held a blue belt or higher, (4) competed regularly over the past 2 years, and (5) had participated in at least one annual competition organized by the International BJJ Federation (IBJJF). Participants were excluded if they had injuries or health conditions that could affect their performance.

The *a priori* sample size was determined based on the data from McCarthy et al. (6), who reported *R*^2^ values ranging from 0.92 to 0.96 (*r* = 0.96–0.98). Using a two-tailed test, α was set at 0.05, statistical power at 0.80, and anticipated effect size at *r* = 0.96. The minimum required sample size was calculated as seven participants using G^*^Power 3.1 (University of Düsseldorf, Germany). Therefore, the sample size of this study (*n* = 22) ensured sufficient power and validity.

This study was conducted in accordance with the ethical principles of the Declaration of Helsinki and was approved by the Institutional Ethics Committee (CODE: BIOPUCV-H 520-2022).

### 2.2. Anthropometric and body composition assessments

Anthropometric and BC measurements were conducted in an exercise and sports science laboratory at a controlled temperature of 21°C during a single session during morning 9:00–12:00 h. Prior to the assessment, participants were instructed to refrain from engaging in strenuous exercise for a minimum of 24 h and to maintain their usual hydration levels to minimize the acute effects on water balance and BC. One week before data collection, all participants received detailed information regarding the procedures, benefits, and potential risks (including X-ray exposure).

Height was measured using a stadiometer (Seca 217, Hamburg, Germany) with 0.1 cm precision, and body mass was measured using a calibrated digital scale (Omron HN300T2) with 0.1 kg precision, following standard protocols. For both measurements, the participants were barefoot and wore light clothing. Global and regional body composition was assessed via DXA using a General Electric Lunar iDXA device (GE Healthcare, Boston, MA, USA; software version 3.6, Lunar DPX, Madison, WI) following the manufacturer's recommendations and NHANES guidelines (16). The participants removed all metallic objects and jewelry and were positioned in a supine recumbent position on the scanning table, wearing only form-fitting underwear. The DXA scanning mode was automatically selected based on body thickness to enhance accuracy and reproducibility. Daily quality control calibrations were performed before each session using a calibration block provided by the manufacturer.

During scanning, the participants remained in the supine position with their (i) head in a neutral position, (ii) arms extended at the sides with space from the torso, (iii) hands flat on the table, and (iv) feet in plantar flexion, parallel, and separated positions. Following ~5 min of rest, the scan was performed between 5 and 10 min (13). Anatomical regions were initially delineated by predetermined DXA system cut lines on the anterior planogram and manually refined according to established protocols (5, 16). The arms, legs, and trunk were differentiated using horizontal lines below the skull and at the level of the iliac crest, vertical lines adjacent to the spine and between the legs, and diagonal lines traversing both glenohumeral joints and femoral necks.

ALM was calculated as the sum of ALM from both arms and legs, according to system software reports. Trunk FFM and total fat mass (FM) were directly extracted from DXA analysis. Segmental body composition metrics, including fat mass percentage (%FM) and BMC, were calculated using standard formulas.

All scans were performed by the same technician certified in x-ray handling by the national health superintendence and by the DXA distributors to minimize inter-operator variability. All required variables were obtained directly from the raw DXA outputs, and the equations were applied as previously published, without local modifications. These models were selected based on their reported high validity (*R*^2^ > 0.90) and common use in both general (6) and athletic populations (13). Table 1 provides the details of the equations used to estimate the SMM.

**Table 1:** Predictive Equations for Skeletal Muscle Mass (SMM) Based on DXA Assessments.

**Study**	**Sample and characteristics**	**DXA scanner (model)**	**Predictive equation for SMM**
McCarthy et al. (6)	475 Caucasian adults (216 men/259 women); mean age ≈ 50 years; BMI ≈ 26 kg·m^2^	QDR 4500A, Hologic	SMM = 1.12 × ALM – 0.63
Kim et al. (5)	270 adults of mixed racial/ethnic background; mean age ≈ 46 years; BMI ≈ 25 kg·m^2^	DPX, Lunar	SMM = 1.18 × ALM – 0.03 × age – 0.14
Sagayama et al. (13)	30 Japanese athletic men; mean age = 19.9 years; BMI = 23.7 kg·m^2^	Discovery A, Hologic	SMM = 1.21 × ALM + 21.85 × (trunk/ALM) – 0.35 × %FM– 18.41

ALM, appendicular lean mass; BMI, body-mass index; DXA, dual-energy X-ray absorptiometry; SMM, skeletal muscle mass;%FM, body-fat percentage; trunk/ALM, trunk-to-appendicular lean-mass ratio.

### 2.3 Statistical analysis

Quantitative data are reported as mean ± standard deviation with 95% confidence intervals (95% CI). Data management was performed using Microsoft Excel v16.37, and all statistical procedures were executed using JASP v0.17 (28). Variable distributions were assessed using skewness–kurtosis coefficients and the Shapiro–Wilk test (17).

The validity of the equations for estimating total SMM in Brazilian Jiu-Jitsu athletes was examined using stepwise multiple linear regression. Sagayama's SMM (13), previously validated in combat sports athletes, served as the dependent variable, whereas the estimates from Kim et al. (5) and McCarthy et al. (6) served as candidate predictors. The coefficient of determination (*R*^2^), root mean square error (RMSE), and regression parameters (slope and intercept) were reported (18).

Additionally, a one-factor repeated-measures ANOVA was used to compare the SMM values derived from the three predictive equations. Sphericity was tested using the Geisser–Greenhouse ε, and when it was violated, the degrees of freedom were adjusted. Pairwise differences were explored using Tukey's *post-hoc* test (17).

Method consistency was quantified using the Pearson product–moment correlation coefficient (r) and interpreted according to the following thresholds: low (0.00–0.30), moderate (0.31–0.49), high (0.50–0.69), very high (0.70–0.89), and nearly perfect to perfect (0.90–1.00) (19). Systematic bias and agreement were evaluated using Bland–Altman plots with limits of agreement (LOA) (20). Relative reliability was assessed using the intraclass correlation coefficient (ICC). The following values were interpreted as follows: poor (>0.50), moderate (>0.50–0.75), good (0.75–0.90), or excellent (0.90–1) (21). Absolute reliability was expressed as the coefficient of variation (CV%), with values < 10% deemed acceptable (22). Statistical significance was set at α = 0.05

## 3 Results

### 3.1 Descriptive results

FFM values were as follows: arms, 7.57 ± 1.68 kg (95% CI: 6.87–8.27 kg); legs, 19.38 ± 3.11 kg (95% CI: 18.08–20.68 kg); trunk, 27.14 ± 3.93 kg (95% CI: 25.50–28.79 kg); and FFM total, 57.83 ± 8.70 kg (95% CI: 54.19–61.46 kg). BMC was 0.47 ± 0.10 kg for arms (95% CI: 0.43–0.51 kg), 0.16 ± 0.36 kg for legs (95% CI: 0.02–0.31 kg), and 3.25 ± 0.51 kg for the whole body (95% CI: 3.04–3.46 kg). The ALM was distributed as follows: arms, 7.11 ± 1.59 kg (95% CI: 6.44–7.77 kg); legs, 18.20 ± 2.94 kg (95% CI: 16.97–19.43 kg); and total ALM, 25.31 ± 4.43 kg (95% CI: 23.45–27.16 kg). Predicted SMM total values were: Kim, 28.95 ± 4.92 kg (95% CI: 26.89–31.00 kg); McCarthy, 27.39 ± 4.96 kg (95% CI: 25.32–29.47 kg); and Sagayama, 27.72 ± 3.71 kg (95% CI: 26.16–29.27 kg) (Table 2).

**Table 2:** Descriptive statistics for total and segmental body composition and skeletal muscle mass estimates.

**Variable (Unit)**	**Mean**	**Standard deviation**	**Lower 95% CI**	**Upper 95% CI**
**Total body composition**				
Body mass (kg)	77.668	9.263	73.797	81.539
Body mass index (kg·m^2^)	26.296	2.677	25.177	27.415
Fat-free mass (kg)	57.827	8.700	54.191	61.462
Fat mass percentage (%)	22.395	6.547	19.660	25.131
Fat mass (kg)	16.620	5.254	14.424	18.816
Bone mineral content total (kg)	3.247	0.507	3.035	3.459
**Segmental body composition**				
Fat-free mass arms (kg)	7.574	1.676	6.874	8.274
Fat-free mass legs (kg)	19.376	3.112	18.076	20.677
Bone mineral content arms (kg)	0.467	0.098	0.426	0.508
Bone mineral content legs (kg)	0.164	0.356	0.015	0.313
Fat-free mass trunk (kg)	27.144	3.927	25.503	28.785
Bone mineral content trunk (kg)	0.268	0.407	0.098	0.438
Appendicular lean mass arms (kg)	7.107	1.587	6.444	7.770
Appendicular lean mass legs (kg)	18.198	2.943	16.968	19.428
Appendicular lean mass total (kg)	25.305	4.432	23.453	27.156
ALM trunk (kg)	26.109	3.799	24.522	27.697
Trunk to ALM ratio	1.040	0.090	1.002	1.077
**Skeletal muscle mass (SMM) total equations**				
SMM Kim (kg)	28.947	4.922	26.890	31.004
SMM McCarthy (kg)	27.394	4.957	25.322	29.465
SMM Sagayama (kg)	27.715	3.713	26.163	29.267

### 3.2 Model Cross-validation

The linear regression model satisfied all key assumptions. Collinearity diagnostics showed no concerns (tolerance = 1.000, VIF = 1.000, condition index = 12.121). Only 0.7% of the explained variance was in the first dimension, and all Cook's distances were below one, indicating no influential case. The Durbin–Watson statistic (2.152; *p* = 0.688) and residual autocorrelation (−0.092) supported independence of errors.

The initial model (without predictors) failed to explain any variability (*R* = 0.000, *R*^2^ = 0.000, and RMSE = 3.713 kg), and the *F* change was zero. Upon inclusion of Kim's SMM, the model achieved a strong multiple correlation (*R* = 0.851; *R*^2^ = 0.724; adjusted *R*^2^ = 0.710), explaining 72.4% of the variance in Sagayama's SMM, with an improved RMSE of 2.000 kg. McCarthy's SMM did not meet the entry criteria (*p* > 0.05) and was excluded.

ANOVA confirmed that the explained variance (SS*R* = 209.548) vastly exceeded the residual variance (SSE = 80.028) out of a total of 289.576 (*F*_1, 20_ = 52.369; *p* < 0.001), confirming the significance of the predictor. The regression intercept (9.139 kg; *t* = 3.512; *p* = 0.002; 95% CI: 2.659–15.831) suggests an implicit baseline SMM, while the slope (0.642 kg·kg^−1^; β = 0.851; *t* = 7.237; *p* < 0.001; 95% CI: 0.429–0.879) indicates that for each additional kilogram estimated by Kim, Sagayama increases by only 0.642 kg, reflecting systematic underestimation.

### 3.3 Content and concurrent validity

**Figure 1:**
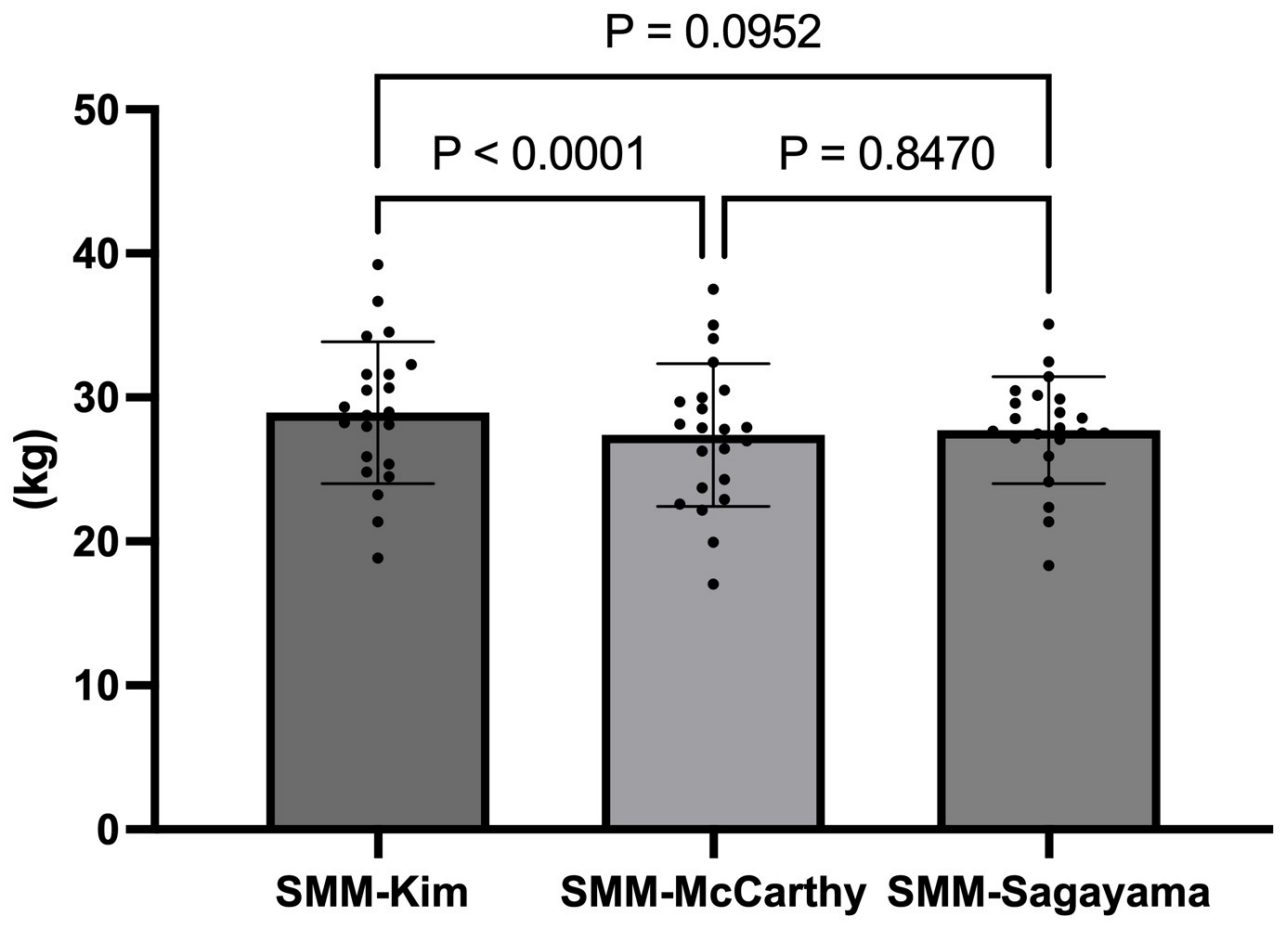
Difference between SMM total estimated between Kim (kg), Sagayama (kg), and McCarthy (kg) equations in Bjj Athletes (*n* = 22).

Repeated-measures ANOVA revealed significant differences between the SMM estimation models (*F*_1.036, 21.77_) = 6.080, *p* = 0.0211, *R*^2^ = 0.225), with the Geisser-Greenhouse correction applied (ε = 0.518). Considerable inter-individual variability was also observed (*F*_21, 42_ = 23.740, *p* < 0.0001, *R*^2^ = 0.902). Pairwise comparisons indicated that the Kim equation produced significantly higher SMM estimates than McCarthy (mean difference = 1.553 kg, 95% CI: 1.322–1.784 kg, *p* < 0.0001), while differences between Sagayama and McCarthy (mean difference = 0.322 kg, 95% CI: −1.149 to 1.792 kg, *p* = 0.847) and between Sagayama and Kim (mean difference = −1.232 kg, 95% CI: −2.645 to 0.182 kg, *p* = 0.0952) were not significant (Figure 1).

### 3.4 Reliability

Relative reliability analyses (CV%) showed the lowest variability with the Sagayama equation (13.4%, 95% CI: 9.44%−17.36%), moderate variability with Kim (17.0%, 95% CI: 11.98%−22.02%), and highest variability with McCarthy (18.1%, 95% CI: 12.74%−23.44%).

Absolute reliability For Kim vs. Sagayama, was ICC = 0.790 (95% CI: 0.564–0.906), while for Sagayama vs. McCarthy, was slightly higher at ICC = 0.810 (95% CI: 0.602–0.916).

### 3.5 Concordance

**Figure 2:**
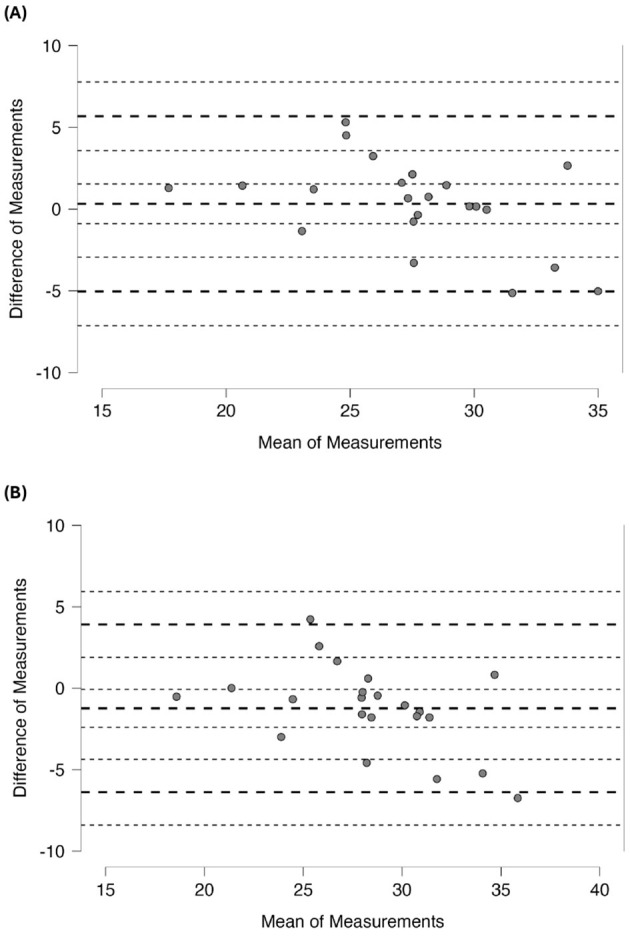
Bland-Altman Plot between SMM equations. **(A)** McCarthy-Sagayama SMM equations, **(B)** Kim-Sagayama SMM equations.

Bland–Altman analyses revealed a systematic bias between Kim and McCarthy mean difference = 1.553 kg, 95% CI: 1.362–1.744 kg; LOA: 0.709 to 2.397 kg, and between Kim and Sagayama mean difference = 1.232 kg, 95% CI: 0.065–2.398 kg; LOA: −3.925 to 6.388 kg. The comparison between McCarthy and Sagayama showed mean difference of −0.321 kg 95% CI: −1.534 to 0.891 kg; LOA −5.683 to 5.040 kg (Figure 2).

## 4 Discussion

This study aimed to examine the validity, reliability, and agreement of SMM predictive equations in BJJ athletes. These findings indicate that the equations proposed by Sagayama et al. have high validity and reliability in this cohort. Nonetheless, linear regression analyses and Bland–Altman plots revealed systematic biases between equations. Specifically, Kim's equation produced significantly higher values than McCarthy's equation (mean difference = 1.553 kg, *p* < 0.001). Sagayama's equation showed the lowest variability (CV%= 13.4%), followed by Kim's (17.0%) and McCarthy's (18.1%), suggesting greater stability for Sagayama's model in this cohort. Given its prior validation in combat sports athletes and its inclusion of variables that more accurately capture morphofunctional characteristics, we hypothesized that Sagayama et al.'s equation would exhibit superior validity.

These results are particularly noteworthy because this study is the first to estimate SMM in BJJ athletes using DXA. These values are partially aligned with those previously reported using anthropometric measurements. Báez et al. (8) examined 25 highly trained athletes, distinguishing between “pass fighters” (*n* = 10; 27.8 ± 5.3 years, 75.0 ± 8.9 kg, 170.0 ± 5.0 cm) and “guard fighters” (*n* = 15; 25.3 ± 5.7 years, 75.9 ± 1.9 kg, 176.5 ± 6.5 cm). Pass guard athletes displayed an absolute SMM of 40.0 ± 4.4 kg (53.0 ± 2.0% body mass), whereas guard fighters presented slightly lower values of 39.3 ± 7.0 kg (51.9 ± 2.2%). The overall sample averaged 39.5 ± 5.8 kg (52.3 ± 2.1%). Detanico et al. (9), using anthropometry in 20 male athletes (28.1 ± 7.1 years), reported a FFM of 63.4 ± 5.8 kg and a FFMI of 20.4 ± 1.4 kg·m^2^, thus providing key body composition benchmarks. Similarly, Monterrosa Quintero et al. (10) evaluated 16 elite athletes (32.2 ± 4.5 years, 80.2 ± 9.7 kg, 180 ± 0.05 cm, 6.3 ± 3 years' experience) and, using validated anthropometric equations, estimated a total SMM of 33.7 ± 3.4 kg (95% CI = 31.9–35.6 kg), underscoring their marked muscular development. Andreato et al. (27), analyzed 15 male athletes (80.3 ± 7.8 kg; 177.5 ± 6.4 cm) and reported a lean body mass of 69.8 ± 4.3 kg and an SMM 47.5 ± 5.8 kg (59.2 ± 5.0% body mass). Pietraszewska et al. (23) used bioelectrical impedance analysis (BIA 101; Akern, Italy) in 49 athletes (77.3 ± 6.5 kg; 177.6 ± 6.4 cm) and recorded an FFM of 65.0 ± 6.4 kg.

In contrast, our DXA-derived SMM values in 22 male BJJ athletes ranged from 27.4 kg to 28.9 kg, which is substantially lower than that reported previously. Báez et al. (8) reported markedly higher anthropometric SMM (39.5 ± 5.8 kg; 52.3% body mass), and Monterrosa Quintero et al. (10) found similarly elevated values (33.7 ± 3.4 kg). Our average DXA-based FFM (57.8 kg) was similar to the anthropometric and BIA estimates reported by Pietraszewska et al. (23) and Detanico et al. (9).

These discrepancies may reflect the methodological differences between studies. DXA offers finer discrimination among adipose, muscle, and bone tissues, whereas anthropometric models often overestimate SMM by including non-contractile tissues. Indeed, BIA may overestimate lean mass due to hydration status.

Bland–Altman analysis revealed systematic estimation biases between the predictive equations. Kim's equation differed most from the Sagayama reference (mean bias = 1.232 kg), whereas McCarthy's equation displayed only a modest bias (0.321 kg). Taken together with previous evidence (13, 26, 29), these findings indicate that the McCarthy and Sagayama equations yield the most accurate SMM estimates for BJJ athletes. Accurate SMM quantification is critical in BJJ, given the distinctive muscular profiles of these athletes compared to sedentary individuals or athletes from other sports as budybuilders and university students (23). BC encompasses lean tissue (muscle, viscera, bone, blood, and lymph) and fat, and accurate differentiation optimizes the monitoring of performance (24, 25). Moreover, Sagayama et al. (13) highlighted that traditional models underestimate SMM in athletes with specific regional muscle distributions, thereby reinforcing the value of sport-specific predictive models.

The convergence of reduced absolute dispersion (CV%) with adequate inter-method consistency (ICC) positions the Sagayama equation as the most methodologically robust option for estimating SMM in BJJ athletes. This superiority is plausibly explained by two complementary factors. First, the Sagayama model was calibrated in combat-sport athletes and explicitly incorporates the trunk-to-appendicular lean-mass ratio—a variable that captures the distinctive regional distribution of musculature in this population. By accounting for such sport-specific morphology, the equation attenuates random error and, consequently, within-subject variability. Second, the narrower confidence limits surrounding both the CV and the ICC indicate greater stability of the estimates, implying that repeated measurements are less susceptible to day-to-day biological and technical noise.

Conversely, the Kim and McCarthy equations—derived from heterogeneous, largely non-athletic samples—exhibited higher coefficients of variation and only moderately strong relative reliability. This pattern suggests that their predictive algorithms do not fully accommodate the elevated and regionally concentrated muscle mass characteristic of grappling athletes, thereby inflating absolute measurement error. Moreover, the discrepancy may stem from the greater variability in skeletal muscle mass estimates produced by these equations, which broadens the spread of values and diminishes longitudinal precision.

Study limitations includes trough despite the *a priori* power analysis, the sample size was relatively small (*n* = 22), which limits its generalizability. The exclusive inclusion of male athletes prevents the direct extrapolation of the results to female athletes, who exhibit distinct hormonal and biomechanical characteristics. Furthermore, direct MRI-based SMM measurements, the gold standard, were not obtained. Although DXA served as the criterion, it can marginally overestimate SMM by including intermuscular fat and other non-contractile tissues. Therefore, the external validity of these equations requires confirmation in BJJ cohorts in the future. Another intrinsic limitation of classic equations is their derivation from the general population, potentially yielding bias in athletes who display unique regional muscle distributions and higher trunk musculature owing to sport-specific adaptations. Sagayama et al. (13) showed that incorporating the trunk to appendicular lean soft tissue ratio substantially reduces estimation error in combat sport athletes. While earlier equations correlated strongly with MRI-measured SMM, they consistently yielded lower mean values than the criterion, underscoring the need to tailor predictive models to combat sports morphology.

Larger and more diverse samples, including females and multiple competitive levels, are recommended, along with functional variables linked to performance and metabolic health. Future studies should validate predictive methods against MRI, integrate functional assessments, and adopt standardized protocols.

Practitioners should carefully select predictive equations; models validated in athletic cohorts and incorporating regional variables, such as Sagayama's equation, are preferable. Combining predictive methods with functional tests provides a comprehensive evaluation of athletic performance.

In summary, convergent evidence confirms that SMM is the predominant component in BJJ athletes, reflecting their demanding physical and technical requirements. However, methodological discrepancies underline the importance of rigorous, sport-specific, and validated protocols for BC assessment to accurately capture this high muscular proportion and inform training and nutrition interventions.

## 5 Conclusion

Although all three equations exhibited accurate validity and reliability, the McCarthy and Sagayama models showed the smallest systematic bias and accurate in estimating SMM in BJJ athletes. These results emphasize the importance of using predictive models specifically calibrated to the morphofunctional characteristics of BJJ athletes.

## Data Availability

The raw data supporting the conclusions of this article will be made available by the authors, without undue reservation.
